# Radiation-Induced Rib Fractures on Magnetic Resonance Imaging Following Proton Therapy for Breast Cancer With Pencil Beam Scanning

**DOI:** 10.7759/cureus.11120

**Published:** 2020-10-24

**Authors:** Ashley R Way, Paul L Wasserman, Raymond Mailhot, Haley Letter

**Affiliations:** 1 Radiology, University of Florida Health Jacksonville, Jacksonville, USA; 2 Radiation Oncology, University of Florida Health Jacksonville, Jacksonville, USA

**Keywords:** pencil beam scanning, radiation induced rib fracture, locally advanced breast-cancer, pathological rib fracture, proton therapy, radiation induced skeletal injury, high risk breast cancer

## Abstract

Radiation-induced rib fractures (RIRF) are long-term complications associated with irradiation of the chest/chest wall. RIRFs are commonly seen in lung- or breast-cancer patients treated with stereotactic body radiotherapy (SBRT) or conventional external beam radiation therapy (EBRT), respectively. We report a case of a 31-year-old female presenting with pathological fractures of the third, fourth, and fifth ribs discovered on magnetic resonance imaging (MRI) as a complication from pencil beam scanning (PBS) proton therapy (PT), of the whole left breast and regional lymph nodes. To our knowledge, this presentation is the first to be initially reported on MRI in radiological literature.

## Introduction

Breast conservation therapy (BCT), which includes lumpectomy and adjuvant radiation therapy (RT) for locally advanced breast cancer (Stage IIb-III), is a valid-alternative to mastectomy [1]. BCT for patients with axillary-nodal disease includes RT of the breast and regional lymph nodes. Proton therapy (PT) is a newer technology, allowing RT to focus on the cancer-target with less dose deposited to surrounding organs/tissue. Doses associated with conventional RT with electron/photons are associated with an increased incidence of cardiotoxicity compared to RT with protons [2]. PT is preferred for left-sided breast cancers to limit cardiac toxicity. Long-term radiation-induced side effects occur in both conventional electron/photon RT or with PT and they include fatigue, local skin changes, lymphedema, radiation-induced metastasis, and/or radiation-induced skeletal injury (RISI).

Radiation-induced rib fractures (RIRF) are a form of RISI [3]. A retrospective study by Peirce et al. reviewed 1624 women with Stage I or II breast cancer treated with conventional (photon and/or electron) external beam radiation therapy (EBRT). The study reported incidence of RIRF in those with a whole breast dose of < 50 Gy was 1.5% versus those treated with > 50 Gy (5.3%), with an overall incidence of RIRF of 1.8% [4]. This is a lower incidence compared to a recent cohort study examining 203 patients receiving PT for breast cancer and reported a 7% RIRF incidence [5,6]. A study by Chia-Chung et al. investigated increases of dose-averaged linear energy transfer (LETd) and relative biological effectiveness (RBE) in the distal edge of the proton beam, due to Bragg peak, as the cause for this increased incidence in those receiving proton RT for breast cancer [6].

RIRFs occur post-RT within the area encompassing the trajectory of the treatment beam, manifesting as dose-dependent late-toxicities from RT of tumors adjacent to, or within close proximity of, the chest wall [7]. A study by Gal et al. observed that osteoblast cell lines irradiated *in vitro* at 0, 2, 4, or 6 Gy demonstrated dose-dependent inhibition of osteoblast proliferation, decreased alkaline phosphatase activity, reduced collagen production, and increased expression of transforming growth factor B1 type I/II receptors [6]. The skeletal matrix can be further compromised in women through use of certain chemotherapy drug types, ovarian-dysfunction, post-menopausal state, and/or metastatic disease-burden; further depleting bone microarchitecture and increasing the risk for RIRF [7]. Women receiving chemotherapy prior to RT had a RIRF risk of 2.3% versus 0.5% treated with RT alone (p<0.0001) [3].

RIRFs can present as painful or non-painful and are typically diagnosed six to 48 months after treatment on follow-up computed tomography (CT) scans [8,9].

## Case presentation

**Figure 1:**
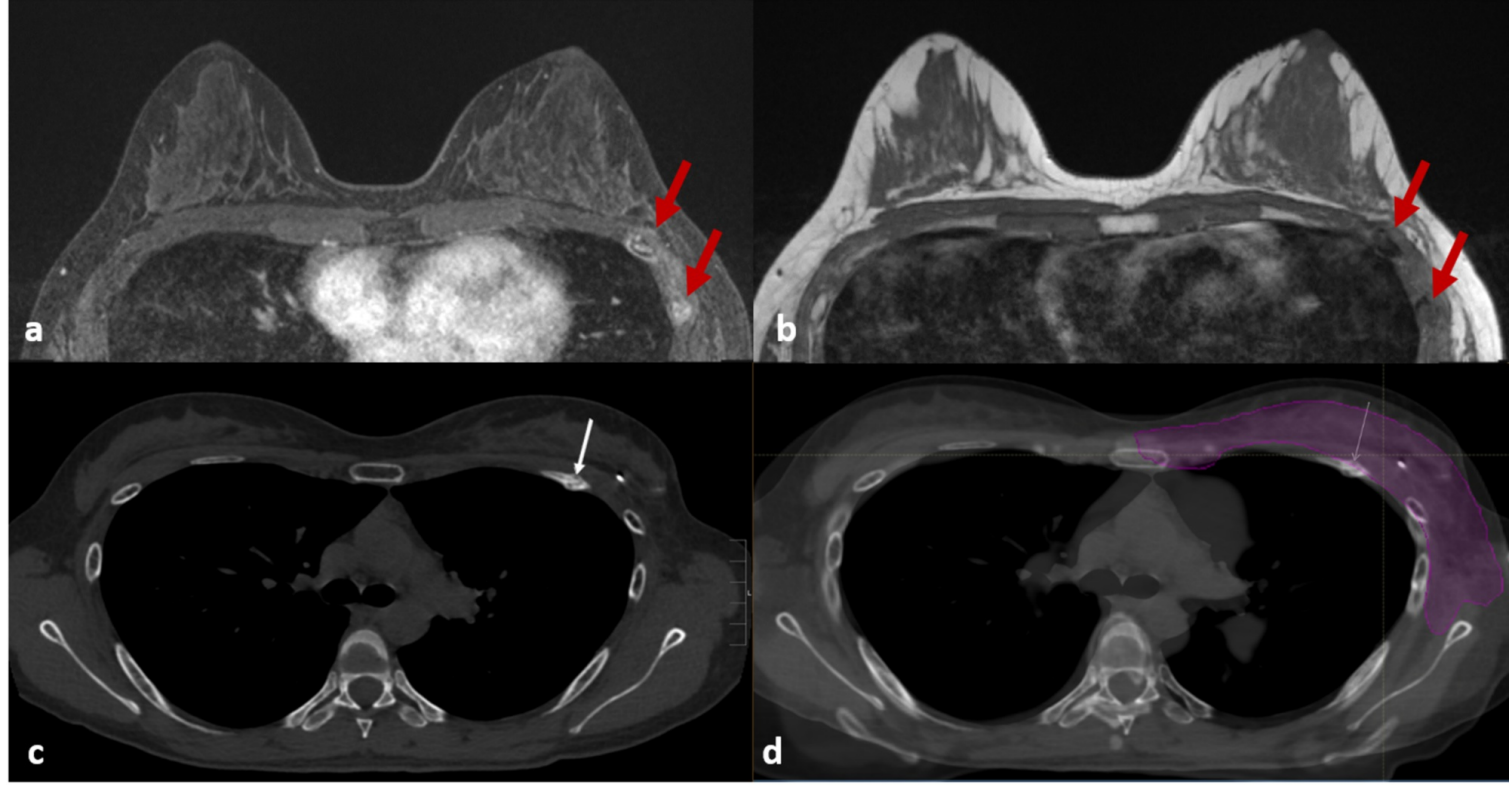
Patient ribs 3, 4, and 5. MRI of the breasts, (a) axial T1 fat saturation post-contrast, (b) axial T1 non-fat saturation; demonstrated focal areas of T1 hypointensity with enhancement of the ribs without adjacent soft tissue abnormality (red arrows) located immediately posterior to the lumpectomy bed and left axilla. Axial CT of the breast, (c) using bone windows demonstrates non-displaced left-sided anterior rib fractures involving the third through fifth ribs (white arrow). CT transposed onto axial slice (d) patient treatment plan; magenta representing the 95% isodose line of the prescription dose.

A 31-year-old patient with history of left breast cancer presented for high-risk screening MRI of the breasts. Two years prior, the patient was diagnosed with stage IIIB (T2 N1 M0) triple-negative left breast cancer with bulky left axillary adenopathy. She underwent neoadjuvant chemotherapy followed by breast conservation therapy with axillary dissection. To limit cardiac toxicity from treatment of the whole left breast and regional lymph nodes, PT was selected rather than conventional photons. The patient tolerated therapy well and had no new complaints at the time of her MRI exam (Figure 1).

## Discussion

The incidence of breast cancer in women under 40 years old is approximately 7% and represents 40% of all cancers found in this age group [10-12]. Women under 40 years old are more likely to have advanced stage cancer (53%) at diagnosis in contrast to women over 65 years old (32%), possibly due to lack of screening prior to age 40 [13]. The five-year survival rate of breast cancer with regional lymph node involvement is 86%, compared to those with cancer localized to only the breast (non-metastatic) (99%) [12,13].

In 2018, the American College of Radiology (ACR) published recommendations for breast cancer screening in women at higher-than-average risk [10]. The ACR identified population subgroups at higher risk as those with genetic predisposition or mutations, women with strong family histories of breast cancer, those treated with chest or mantle RT at a very young age, African American race, as well as those women with a past history of breast cancer who were diagnosed prior to age 50 [10].

Unfortunately, there is paucity of information regarding descriptive features of RIRF on MRI. The current ACR recommendations now indicate the use of MRI for yearly follow-up in high-risk groups, like our patient. Due to this change, it is possible that the frequency of diagnosis of RIRF from both PT and conventional RT on MRI will increase, compared to historical presentation on CT. It is important for radiologist to be aware of these complications and their features on MRI.

## Conclusions

The current recommendation from the American College of Radiology is for annual MRI screening of the breasts, in addition to annual mammography, for women with past history of breast-cancer diagnosed before age 50. While MRI demonstrates superior soft tissue resolution compared to CT, it is inferior to CT for evaluation of osseous structures. Pathologic rib fracture from metastatic disease is the most important differential diagnostic consideration and must be excluded. Therefore, CT and MRI play complimentary roles in evaluating rib fractures in a patient with past history of radiation. Given the current ACR screening guidelines, incidental detection of RIRF may become more prevalent on MRI.

## References

[1] Alm El-Din MA, Taghian AG (2009). Breast conservation therapy for patients with locally advanced breast cancer. Semin Radiat Oncol.

[2] Benveniste MF, Gomez D, Carter BW (2019). Recognizing radiation therapy-related complications in the chest. RadioGraphics.

[3] Engleman MA, Woloschak G, Small W (2006). Radiation-induced skeletal injury. Radiation Toxicity: A Practical Guide.

[4] Pierce SM, Recht A, Lingos TI (1992). Long-term radiation complications following conservative surgery (CS) and radiation therapy (RT) in patients with early stage breast cancer. Int J Radiat Oncol Biol Phys.

[5] Ottanelli S (2015). Prevention and treatment of bone fragility in cancer patient. Clin Cases Miner Bone Metab.

[6] Wang CC, McNamara AL, Shin J (2020). End-of-range radiobiological effect on rib fractures in patients receiving proton therapy for breast cancer. Int J Radiat Oncol Biol Phys.

[7] Meric F, Buchholz TA, Mirza NQ (2002). Long-term complications associated with breast-conservation surgery and radiotherapy. Ann Surg Oncol.

[8] Andolino DL, Forquer JA, Henderson MA (2011). Chest wall toxicity after stereotactic body radiotherapy for malignant lesions of the lung and liver. Int J Radiat Oncol Biol Phys.

[9] Wijsman R, Braam PM, Bussink J (2017). Radiation-induced rib fractures after stereotactic body radiation therapy: predict to prevent?. Radiother Oncol.

[10] Monticciolo DL, Newell MS, Moy L (2018). Breast cancer screening in women at higher-than-average risk: Recommendations from the ACR. J Am Coll Radiol.

[11] Harris SR (2016). Differentiating the causes of spontaneous rib fracture after breast cancer. Clin Breast Cancer.

[12] Anders CK, Johnson R, Litton J (2009). Breast cancer before age 40 years. Semin Oncol.

[13] (2020). Cancer Facts & Figures 2020. https://www.cancer.org/research/cancer-facts-statistics/all-cancer-facts-figures/cancer-facts-figures-2020.html.

